# Reported Xylazine Use Among Adults Aged ≥18 Years Evaluated for Substance Use Treatment — United States, July 2022–September 2023

**DOI:** 10.15585/mmwr.mm7326a2

**Published:** 2024-07-04

**Authors:** Xinyi Jiang, Sarah Connolly, Andrea E. Strahan, Liz Rivera Blanco, Christina A. Mikosz, Gery P. Guy, Deborah Dowell

**Affiliations:** ^1^Division of Overdose Prevention, National Center for Injury Prevention and Control, CDC; ^2^Epidemic Intelligence Service, CDC.

**Summary**

**What is already known about this topic?**

Xylazine, a nonopioid sedative, has been increasingly detected in illegally manufactured fentanyl (IMF) and IMF-involved U.S. overdose deaths; most xylazine-involved overdose deaths involve IMF.

**What is added by this report?**

Among adults evaluated for substance use treatment and reporting past–30-day IMF or heroin use or IMF or heroin as their primary lifetime substance use problem, those also reporting xylazine use reported more past nonfatal overdoses, and higher percentages of persons who reported xylazine use reported other recent substance use and polysubstance use than did persons who did not report xylazine use.

**What are the implications for public health practice?**

Provision of nonjudgmental care and services and linkage to and retention in effective substance use treatment might reduce harms, including overdose among persons reporting xylazine use.

## Abstract

Xylazine has been increasingly detected in illegally manufactured fentanyl (IMF) products and overdose deaths in the United States; most xylazine-involved overdose deaths involve IMF. A convenience sample of U.S. adults aged ≥18 years was identified from those evaluated for substance use treatment during July 2022–September 2023. Data were collected using the Addiction Severity Index-Multimedia Version clinical assessment tool. Among 43,947 adults, 6,415 (14.6%) reported IMF or heroin as their primary lifetime substance-use problem; 5,344 (12.2%) reported recent (i.e., past–30-day) IMF or heroin use. Among adults reporting IMF or heroin as their primary lifetime substance-use problem, 817 (12.7%) reported ever using xylazine. Among adults reporting recent IMF or heroin use, 443 (8.3%) reported recent xylazine use. Among adults reporting IMF or heroin use recently or as their primary lifetime substance-use problem, those reporting xylazine use reported a median of two past nonfatal overdoses from any drug compared with a median of one overdose among those who did not report xylazine use; as well, higher percentages of persons who reported xylazine use reported other recent substance use and polysubstance use. Provision of nonjudgmental care and services, including naloxone, wound care, and linkage to and retention of persons in effective substance use treatment, might reduce harms including overdose among persons reporting xylazine use.

## Introduction

Xylazine, a nonopioid sedative, has been increasingly detected in illegally manufactured fentanyl (IMF) products* and in U.S. overdose deaths (*1*). Most detected xylazine-involved overdose deaths also involve IMF (*2*); IMF-involved deaths with xylazine detected rose 276% in 21 jurisdictions during January 2019– June 2022 (*3*). Drugs sold as heroin increasingly contain IMF, which could lead persons seeking heroin to consume IMF, either knowingly or unknowingly (*4*,*5*). Xylazine has also been associated with skin lesions (*6*) that appear to be independent of the route of xylazine administration.^†^ To guide the development and implementation of prevention and response efforts, a cross-sectional U.S. study examining characteristics of adults evaluated for substance use treatment who reported IMF or heroin use and who also responded to questions about xylazine use was conducted.

## Methods

### Data Source

A convenience sample of U.S. adults aged ≥18 years evaluated for substance use treatment was identified using the validated, self-administered Addiction Severity Index-Multimedia Version (ASI-MV) clinical assessment tool^§^ during July 2022–September 2023 (*7*). The ASI-MV tool, an instrument integral to the National Addictions Vigilance Intervention and Prevention Program (NAVIPPRO) and used for clinical treatment planning and triage purposes, is administered at substance-use treatment and other facilities or programs (including criminal justice programs, drug courts, and homeless services) and collects information on demographic characteristics, substance use patterns, routes of drug administration, and overdose history. Data were obtained from responses to the NAVIPPRO ASI-MV tool. Adults included in the analysis were those who responded to questions about xylazine use among those who reported IMF or heroin use. Xylazine was identified in ASI-MV as “xylazine (sometimes combined with heroin, fentanyl, or cocaine; sometimes known as sleep cut/tranq dope/Anestesia de Caballo).”

### Statistical Analysis

Wilcoxon rank-sum tests for continuous variables and Pearson’s chi-square tests for categorical variables^¶^ were used to compare the distribution of demographic characteristics for the following groups: 1) adults reporting ever versus never using xylazine, among adults reporting IMF or heroin as their primary lifetime substance use problem**; and 2) adults reporting recent (i.e., past–30-day) xylazine use^††^ versus no recent xylazine use,^§§^ among adults reporting recent IMF or heroin use. P-values <0.05 (two-sided) were considered statistically significant. Analyses were conducted using SAS (version 9.4: SAS Institute). This activity was reviewed by CDC, deemed not research, and was conducted consistent with applicable federal law and CDC policy.^¶¶^

## Results

### Study Participants

Among 45,015 unique adults who completed assessments during the study period, 31,675 (70.4%) were assessed in the southern United States; 1,068 (2.4%) persons were excluded because they did not respond to the xylazine questions.*** Among the remaining 43,947 respondents, 6,415 (14.6%) reported IMF or heroin as their primary lifetime substance use problem, and 5,344 (12.2%) reported recent IMF or heroin use.^†††^ Among all 43,947 respondents, a total of 1,924 (4.4%) reported ever using xylazine.

### Xylazine Use Among Adults Reporting IMF or Heroin as Their Primary Lifetime Substance-Use Problem

Among 6,415 adults reporting IMF or heroin as their primary lifetime substance-use problem, 817 (12.7%) reported ever using xylazine. The median age of persons in this group was 34.0 years (IQR = 29.0–40.0 years); most were male (60.7%), non-Hispanic White (78.0%), had a high school education or less (73.5%), and had ever started treatment for substance use (77.6%) (Table). No statistically significant differences in age, sex, education, primary substance-use problem, or history of starting treatment for substance use were identified among adults who reported ever versus never using xylazine; however, a higher percentage of persons who ever used xylazine reported using at least one other substance recently (p<0.01). Further, the percentage of those who reported polysubstance use (use of two or more other substances apart from IMF or heroin and xylazine)^§§§^ during the preceding 30 days was higher among those who had ever used xylazine (57.2%) than that among those who had never used it (36.1%) (p<0.01). Those reporting ever using xylazine reported more lifetime nonfatal overdoses from any drug (median = two) than did those who never used xylazine (median = one) (p<0.01).

**TABLE:** Characteristics of adults evaluated for substance use treatment, by reported xylazine use — United States, July 2022–September 2023

Characteristic	No. (%)					
	Xylazine use among adults reporting IMF or heroin as their primary lifetime substance-use problem n = 6,415			Past–30-day xylazine use among adults reporting past–30-day IMF or heroin use n = 5,344*		
	Ever used n = 817 (12.7%)	Never used n = 5,598 (87.3%)	p-value	Yes n = 443 (8.3%)	No n = 4,896 (91.6%)	p-value
**Median age, yrs (IQR)**	34.0 (29.0–40.0)	34.0 (29.0–40.0)	0.21	34.0 (30.0–41.0)	34.0 (29.0–41.0)	0.23
**Sex**						
Female	319 (39.1)	2,005 (35.8)	0.07	186 (42.0)	1,700 (34.7)	<0.01
Male	496 (60.7)	3,592 (64.2)		256 (57.8)	3,194 (65.2)	
Unknown/No response	2 (0.2)	1 (0.02)		1 (0.2)	2 (0.0)	
**Race and ethnicity**						
AI/AN, NH	16 (2.0)	194 (3.5)	<0.01	12 (2.7)	168 (3.4)	0.03
Black or African American, NH	51 (6.2)	536 (9.6)		32 (7.2)	502 (10.3)	
White, NH	637 (78.0)	4,080 (72.9)		343 (77.4)	3,478 (71.0)	
Hispanic or Latino	60 (7.3)	494 (8.8)		28 (6.3)	462 (9.4)	
Other, NH	53 (6.5)	294 (5.3)		28 (6.3)	286 (5.8)	
**Highest education level achieved**						
Less than high school	204 (25.0)	1,326 (23.7)	0.41	100 (22.6)	1,208 (24.7)	0.59
High school	396 (48.5)	2,848 (50.9)		227 (51.2)	2,472 (50.5)	
Any college	217 (26.6)	1,415 (25.3)		115 (26.0)	1,207 (24.7)	
Unknown/No response	0 (—)	9 (0.16)		1 (0.2)	9 (0.2)	
**Entered treatment for substance use (e.g., alcohol or drugs or both) during lifetime**						
Yes	634 (77.6)	4,365 (78.0)	0.65	318 (71.8)	3,663 (74.8)	0.12
No	116 (14.2)	839 (15.0)		85 (19.2)	803 (16.4)	
Unknown/No response	67 (8.2)	394 (7.0)		40 (9.0)	430 (8.8)	
**Route of xylazine use** ^†^						
Injection drug use	259 (31.7)	NA	NA	136 (30.7)	NA	NA
No injection drug use	478 (58.5)	NA		289 (65.2)	NA	
Unknown/No response	80 (9.8)	NA		18 (4.1)	NA	
**Substance use in the past 30 days (i.e., recent substance use)**						
Alcohol	222 (27.2)	1,201 (21.5)	<0.01	200 (45.2)	1,753 (35.8)	<0.01
Cannabis^§^	307 (37.6)	1,292 (23.1)	<0.01	248 (56.0)	1,777 (36.3)	<0.01
Cocaine or crack	166 (20.3)	721 (12.9)	<0.01	173 (39.1)	1,190 (24.3)	<0.01
Heroin	371 (45.4)	1,722 (30.8)	<0.01	338 (76.3)	2,497 (51.0)	<0.01
Illegal stimulant^¶^	300 (36.7)	1,086 (19.4)	<0.01	268 (60.5)	1,718 (35.1)	<0.01
IMF	495 (60.6)	2,874 (51.3)	<0.01	419 (94.6)	4,073 (83.2)	<0.01
Prescription opioid misuse**	518 (63.4)	2,006 (35.8)	<0.01	333 (75.2)	2,038 (41.6)	<0.01
Prescription sedative, tranquilizer, or sleeping pill^††^	174 (21.3)	721 (12.9)	<0.01	159 (35.9)	995 (20.3)	<0.01
Prescription stimulant misuse^§§^	37 (4.5)	83 (1.5)	<0.01	50 (11.3)	135 (2.8)	<0.01
Xylazine	326 (39.9)	0 (—)	<0.01	443 (100.0)	0 (—)	<0.01
Other substances^¶¶^	148 (18.1)	221 (4.0)	<0.01	151 (34.1)	405 (8.3)	<0.01
Past–30-day polysubstance use (use of ≥2 other substances, apart from IMF or heroin and xylazine)***						
Yes	467 (57.2)	2,020 (36.1)	<0.01	376 (84.9)	2,832 (57.8)	<0.01
No	350 (42.8)	3,578 (63.9)		67 (15.1)	2,064 (42.2)	
**Median no. of other substances used in the past 30 days, (IQR)** ^†††^	2.0 (1.0–4.0)	1.0 (0.0–2.0)	<0.01	3.0 (2.0–5.0)	2.0 (1.0–3.0)	<0.01
**Primary substance use problem**						
Heroin	332 (40.6)	2,334 (41.7)	0.57	97 (21.9)	1,051 (21.5)	0.65
IMF	485 (59.4)	3,264 (58.3)		217 (49.0)	2,566 (52.4)	
Other substances or none	0 (—)	0 (—)		117 (26.4)	1,256 (25.7)	
Unknown/No response	0 (—)	0 (—)		12 (2.7)	23 (0.5)	
**Median no. of lifetime nonfatal overdoses related to any drug, (IQR)** ^§§§ ^	2.0 (0.0–5.0)	1.0 (0.0–4.0)	<0.01	2.0 (0.0–5.0)	1.0 (0.0–4.0)	<0.01

**Source:** The National Addictions Vigilance Intervention and Prevention Program Addiction Severity Index-Multimedia Version data sets July 2022–September 2023. The unit of analysis was each adult.

**Abbreviations:** AI/AN = American Indian or Alaska Native; IMF = illegally manufactured fentanyl; NA = not applicable; NH = non-Hispanic.

* Among 5,344 adults, 5 (0.1%) did not respond to question related “xylazine use in the past 30-day.” “No xylazine use past 30 days” includes adults who never used xylazine or those who used xylazine >30 days ago. Days of xylazine use and use of IMF, heroin, or both in the past 30 days might or might not overlap.

^†^ For the “no injection drug use” category, swallowed, snorted or sniffed, or smoked was reported. For the “injection drug use” category, swallowed, snorted or sniffed, or smoked might also be reported.

^§^ Cannabis is defined as marijuana, hashish, or a prescription cannabinoid product (e.g., Cesamet or Marinol).

^¶^ Illegal stimulant included bath salts, ecstasy, illegal methamphetamines, or a combination of these.

** Prescription opioids include selection of past–30-day misuse of one or more prescription opioid medications, such as Oxycontin, oxycodone, Vicodin, Percocet, methadone, or buprenorphine. Prescription opioid misuse is any use that is not considered “use as prescribed.” For prescription opioids, “use as prescribed” requires 1) having a current pain problem and taking a prescribed opioid medication for pain during the past 30 days, 2) obtaining the medication only from one’s own prescription, and 3) no use of the medication via any route of administration other than what was prescribed. Regarding the source of past–30-day misused prescription opioids among “xylazine use past 30 days“ category (333), “own prescription” was the most common source (184; 55.3%), followed by “dealer” (159; 47.8%), “family/friend” (119; 35.7%), “other source” (105; 31.5%), “stolen” (28; 8.4%), “internet” (13; 3.90%), and “prescription forgery” (10; 3.0%). Sources of procurement were not mutually exclusive.

^†† ^Prescription sedatives, tranquilizers, or sleeping pills, such as Ambien, Klonopin, Lunesta, Valium, or Xanax, might be misused or used as prescribed.

^§§^ Prescription stimulants include selection of past–30-day misuse of prescription stimulant medications, such as amphetamine (e.g., Adderall or Vyvanse) or methylphenidate (e.g., Concerta, Focalin, or Ritalin). Prescription stimulant misuse is any use that is not considered “use as prescribed.” For prescription stimulants, “use as prescribed” is defined as obtaining the stimulant medication only from one’s own prescription and no use of the medication via any route of administration other than what was prescribed. Misuse is also assigned if a respondent indicates having used the medication during the past 30 days, “not in a way prescribed by your doctor to treat a diagnosed attention deficit or hyperactivity disorder.”

^¶¶^ Other substances included barbiturates such as phenobarbital, Seconal, and Fiorinal (barbs, reds, or downers); hallucinogens like LSD or acid, PCP, mushrooms, or angel dust; inhalants like glue, paint, gasoline, or nitrous oxide; GHB (G, Liquid G, Liquid X, or Fantasy); ketamine (K, Special K, or Vitamin K); K2 (spice or synthetic cannabis); Rohypnol (Roche, Roofies, or Rope); over-the-counter medication, such as cough medicine, taken not as directed; and other (or unknown).

*** Past–30-day polysubstance use includes past–30-day use (or prescription medication misuse) of at least two of the following substances: alcohol; cannabis; cocaine or crack; prescription opioid (misuse); prescription stimulant (misuse); illegal stimulant use; prescription sedatives, tranquilizers, or sleeping pills use; barbiturates; hallucinogens; inhalants; GHB; ketamine; K2; Rohypnol; over-the-counter medications; and other unspecified drugs. Polysubstance use here does not necessarily represent use of substances simultaneously.

^†††^ Excluding IMF, heroin, and xylazine. Other substances include alcohol; cannabis; cocaine or crack; prescription opioid (misuse); prescription stimulant (misuse); illegal stimulant use; prescription sedatives, tranquilizers, or sleeping pills use; barbiturates; hallucinogens; inhalants; GHB; ketamine; K2; Rohypnol; over-the-counter medications; and other unspecified drugs.

^§§§^ The sample size is 815 among the “Ever used xylazine” category, 5,570 among the “Never used xylazine” category, 430 among the “Xylazine use past 30 days” category, and 4,849 among the “No xylazine use past 30 days” category.

The most common substances used during the preceding 30 days by persons reporting ever using xylazine were misused prescription opioids (63.4%),^¶¶¶^^,^**** IMF (60.6%), and heroin (45.4%). Recent prescription opioid misuse was nearly twice as common among those who reported ever (63.4%) versus never (35.8%) using xylazine. The majority of respondents (58.5%) reported using xylazine by swallowing, snorting or sniffing, or smoking, without injection; nearly one third (31.7%) reported injecting xylazine.

### Xylazine Use Among Adults Reporting Recent IMF or Heroin Use

Among 5,344 adults reporting recent (past–30-day) IMF or heroin use, 443 (8.3%) reported past–30-day xylazine use (Table). Compared with the percentage of adults who reported no recent xylazine use, higher percentages of persons with recent xylazine use were female (42.0% versus 34.7%; p<0.01) and reported other recent polysubstance use (84.9% versus 57.8%; p<0.01). Persons reporting recent xylazine use reported a median of two lifetime nonfatal overdoses from any drug compared with a median of one among those without recent xylazine use (p<0.01). Most persons (65.2%) reported using xylazine by swallowing, snorting or sniffing, or smoking, without injection; 30.7% reported injecting xylazine. Among persons reporting recent IMF or heroin use, a higher percentage of those reporting recent xylazine use reported other recent substance use (range = 11.3% [prescription stimulant misuse] to 94.6% [IMF]) than did persons who did not report recent xylazine use (range = 2.8% [prescription stimulant misuse] to 83.2% [IMF]) (all p-values <0.01).

## Discussion

Among adults evaluated for substance use treatment and reporting IMF or heroin use during the past 30 days or as their primary lifetime substance use problem, those reporting xylazine use reported more past nonfatal overdoses; as well higher percentages of persons who reported xylazine use reported other recent substance use and polysubstance use than did those who did not report xylazine use. Whether xylazine increases IMF-involved overdose risk is not clear (*5*); a previous analysis found that overdose circumstances and other drug co-involvement were largely similar between IMF-involved deaths with and without xylazine detected (*3*). Reported xylazine exposure might be associated with higher numbers of nonfatal overdoses because both nonfatal overdoses (*8*) and xylazine exposure are associated with polysubstance use, consistent with the finding in this report. Although naloxone cannot reverse the effects of xylazine, its distribution should be expanded because xylazine is most commonly combined with IMF, the effects of which do respond to naloxone (*3*,*5*,*9*). More research is needed to understand whether xylazine use might indicate other polysubstance use or might be associated with unique withdrawal and dependence syndromes and the implications for treatment of concomitant opioid use disorder (*5*,*9*).

Data to guide development and implementation of recommendations for treatment of persons with xylazine use are limited; however, recent reviews have noted that persons with extensive xylazine-associated skin involvement might experience additional difficulties accessing needed services, including substance use treatment and wound care, which can, in turn, exacerbate substance use and interfere with wound healing (*5*,*9*). Reducing stigma associated with substance use, and specifically with wounds as a sign of substance use, is an important component of linking and retaining persons with substance-associated skin involvement in low-barrier substance use treatment programs as a means to help them access harm reduction services and wound care (*5*). Parameters for use and timing of clinical testing for xylazine use have not been clearly defined (*5*); however, persons who use illicit drugs, including opioids, cocaine or crack, or amphetamine-type drugs, have reported a desire for xylazine test strips (*10*). Expanding clinical care and drug checking programs (including, for example, provision of fentanyl and xylazine test strips) might facilitate engagement of persons who use drugs in harm reduction services and help them limit the potential for associated harms.

### Limitations

The findings in this report are subject to at least seven limitations. First, xylazine use could be under- or overreported. Information was not available concerning the validity of self-reported xylazine use, including whether presence of xylazine was confirmed with test strips or whether respondents believed they consumed xylazine. Of note, xylazine or IMF is often mixed with other substances, and respondents might have been unaware of their exposures (*2*,*3*,*10*), potentially leading to underreporting of actual xylazine or IMF exposure. Second, ASI-MV data are self-reported and subject to social desirability, reporting biases, and recall error. Third, persons who use multiple substances might be more likely to have heard of xylazine and, therefore, more likely to report using xylazine. Fourth, the ASI-MV does not ask whether persons intentionally used xylazine or were simply exposed to it as an adulterant of other drugs. A survey of persons who inject drugs in Philadelphia found that whereas some persons might seek xylazine, most prefer not to use xylazine (*4*). This preference might be due to concerns about negative effects of xylazine, such as worsened withdrawal symptoms and wounds (*2*,*4*). Fifth, for each substance used, no start and end use dates were recorded; thus, identifying adults using xylazine and other substances simultaneously was not possible. Sixth, because of the study design, xylazine use without IMF or heroin (e.g., xylazine used with cocaine) was not reported. Future study is needed to explore the characteristics of this population and help them mitigate any associated harms. Finally, data are a convenience sample, and most assessments came from the southern United States; xylazine presence varies in the illicit drug supply across the United States.^††††^ Thus, results might not be generalizable to all U.S. adults being assessed for substance use treatment.

### Implications for Public Health Practice

Most fatal overdoses involving xylazine in the United States also involve IMF (*2*); to reduce fatal overdoses, linking and retaining persons who use xylazine in effective substance use treatment, including medications for opioid use disorder as indicated, and expanding naloxone distribution are critical. To help engage persons who use drugs in treatment, mitigate harms of drug use, and build trust among persons not yet ready for substance use treatment, jurisdictions can expand access to harm reduction services, including xylazine test strips and wound care. Broader interventions are needed to reduce stigma directed toward persons who use drugs and increase awareness of their treatment and service needs so that services can be accessed without judgment.

## Footnotes

* Pharmaceutical fentanyl is a highly potent (50–100 times more potent than morphine) synthetic opioid indicated for the treatment of severe pain. IMF is produced and sold through illegal drug markets and is often mixed with heroin or cocaine. Information on fentanyl overdose prevention is available: https://www.cdc.gov/overdose-prevention/about/fentanyl.html?CDC_AAref_Val = https://www.cdc.gov/opioids/basics/fentanyl.html

†
https://hip.phila.gov/document/3154/PDPH-HAN_Update_13_Xylazine_12.08.2022.pdf/

§ Adults could be assessed by ASI-MV multiple times. To analyze data at the person level, for adults with multiple assessments, the most recent assessment was selected if multiple assessments were performed on the same day; for adults with multiple assessments collected on different days, the earliest assessment was selected.

¶ The “unknown” or “no response” categories for each demographic characteristic and all variables related to substance use were excluded from the Wilcoxon rank-sum tests and Pearson’s chi-square tests.

** NAVIPPRO defined the primary lifetime substance use problem as the primary or most serious problem persons reported among the substances they used in their lifetime (only one substance could be selected). The definitions of IMF and heroin in the ASI-MV were “Street fentanyl (illegal fentanyl, carfentanil – sometimes combined with other drugs such as heroin or cocaine),” and “heroin,” respectively.

†† Days of xylazine use and IMF or heroin use might or might not overlap.

§§ The no recent xylazine use category includes adults who never used xylazine or those who last used xylazine >30 days earlier.

¶¶ 45 C.F.R. part 46, 21 C.F.R. part 56; 42 U.S.C. Sect. 241(d); 5 U.S.C. Sect. 552a; 44 U.S.C. Sect. 3501 et seq.

*** During the study period, 48,872 assessments were completed by 45,015 unique persons from 358 sites located in 32 states (7.8% of assessments were repeat assessments, meaning that they were completed by a person who had already completed one assessment). Among the 45,015 unique persons, 2,602 (5.8%) were assessed in the Northeast, 4,427 (9.8%) in the Midwest, 31,675 (70.4%) in the South, and 6,311 (14.0%) in the West. Overall, 1,068 unique adults did not respond to the xylazine question and were excluded.

††† The 5,344 persons who reported recent IMF or heroin use included 3,936 (73.7%) persons who reported IMF or heroin as their primary lifetime substance use problem. An additional 1,408 (26.3%) persons reported recent IMF or heroin use but did not report IMF or heroin as their primary substance use problem.

§§§ Polysubstance use (apart from IMF or heroin and xylazine) includes past–30-day use (or prescription medication misuse) of at least two of the following: alcohol; cannabis; cocaine or crack; prescription opioid (misuse); prescription stimulant (misuse); illegal stimulant use; prescription sedatives, tranquilizers, or sleeping pills use; barbiturates; hallucinogens; inhalants; GHB; ketamine; K2; Rohypnol; over-the-counter medications; and other unspecified drugs. Polysubstance use here does not necessarily represent use of substances simultaneously.

¶¶¶ Prescription opioid misuse is any use that is not considered “use as prescribed.” For prescription opioids, “use as prescribed” requires 1) having a current pain problem and taking a prescribed opioid medication for pain during the past 30 days; 2) obtaining the medication only using one’s own prescription; and 3) no use of the medication via any route of administration other than that prescribed.

**** Nonmutually exclusive sources of past–30-day misused prescription opioids among 518 adults reporting primary IMF or heroin use and any xylazine use were “own prescription” (332; 64.1%), “dealer” (178; 34.4%), “family/friend” (149; 28.8%), “other source” (119; 23.0%), “stolen” (21; 4.1%), “internet” (9; 1.7%), and “prescription forgery” (6; 1.2%).

†††† https://www.dea.gov/sites/default/files/2022-12/The%20Growing%20Threat%20of%20Xylazine%20and%20its%20Mixture%20with%20Illicit%20Drugs.pdf; https://www.muni.org/Departments/Assembly/Documents/Webpage%20-%20Health%20Policy%20Committee/VOA%20Alaska%20-%20Millennium%20Health%20Signals%20Report%20Xylazine.pdf
